# Glutathione: Lights and Shadows in Cancer Patients

**DOI:** 10.3390/biomedicines11082226

**Published:** 2023-08-08

**Authors:** Herbert Ryan Marini, Bianca Arianna Facchini, Raffaele di Francia, José Freni, Domenico Puzzolo, Liliana Montella, Gaetano Facchini, Alessandro Ottaiano, Massimiliano Berretta, Letteria Minutoli

**Affiliations:** 1 Department of Clinical and Experimental Medicine, University of Messina, 98125 Messina, Italy; hrmarini@unime.it (H.R.M.); lminutoli@unime.it (L.M.); 2 Department of Precision Medicine, University of Campania “Luigi Vanvitelli”, 80133 Napoli, Italy; biancaarianna.facchini@studenti.unicampania.it; 3 Gruppo Oncologico Ricercatori Italiani (GORI-ONLUS), 33170 Pordenone, Italy; rdifrancia@iapharmagen.com; 4 Department of Biomedical and Dental Sciences and Morphofunctional Imaging, University of Messina, 98125 Messina, Italy; jose.freni@unime.it (J.F.); puzzolo@unime.it (D.P.); 5 Division of Medical Oncology, “Santa Maria delle Grazie” Hospital, ASL Napoli 2 Nord, 80078 Pozzuoli, Italy; liliana.montella@aslnapoli2nord.it (L.M.); gaetano.facchini@aslnapoli2nord.it (G.F.); 6 Istituto Nazionale Tumori di Napoli, IRCCS “G. Pascale”, 80131 Napoli, Italy; a.ottaiano@istitutotumori.na.it

**Keywords:** glutathione, cancer, antioxidants, toxicity, diet, nutraceuticals, chemotherapy

## Abstract

In cases of cellular injury, there is an observed increase in the production of reactive oxygen species (ROS). When this production becomes excessive, it can result in various conditions, including cancerogenesis. Glutathione (GSH), the most abundant thiol-containing antioxidant, is fundamental to re-establishing redox homeostasis. In order to evaluate the role of GSH and its antioxi-dant effects in patients affected by cancer, we performed a thorough search on Medline and EMBASE databases for relevant clinical and/or preclinical studies, with particular regard to diet, toxicities, and pharmacological processes. The conjugation of GSH with xenobiotics, including anti-cancer drugs, can result in either of two effects: xenobiotics may lose their harmful effects, or GSH conjugation may enhance their toxicity by inducing bioactivation. While being an interesting weapon against chemotherapy-induced toxicities, GSH may also have a potential protective role for cancer cells. New studies are necessary to better explain the relationship between GSH and cancer. Although self-prescribed glutathione (GSH) implementation is prevalent among cancer patients with the intention of reducing the toxic effects of anticancer treatments and potentially preventing damage to normal tissues, this belief lacks substantial scientific evidence for its efficacy in reducing toxicity, except in the case of cisplatin-related neurotoxicity. Therefore, the use of GSH should only be considered under medical supervision, taking into account the appropriate timing and setting.

## 1. Introduction

Glutathione (GSH) is a tripeptide molecule consisting of the amino acids L-glutamate (Glu), cysteine (Cys), and glycine (Gly), and represents one of the most abundant antioxidants in human cells. It plays a fundamental role in counterattacking the overproduction of reactive oxygen species (ROS) [1] that often occurs under cell injury conditions and that may lead, when uncontrolled, to the development of several types of diseases, including cancer [2]. 

This well-known antioxidant effect has led during the past decade to an increased diffusion of GSH-based self-prescribed implementation by patients, aiming to prevent cell injury possibly leading to cancer onset or to reduce anticancer treatments’ toxicities when cancer has already been diagnosed. 

In this review, we will assess what is currently known about the metabolism of GSH and its role in cell homeostasis, as well as its possible effects on cancer cells and on the efficacy of cancer treatments.

## 2. Materials and Methods 

**Figure 1:**
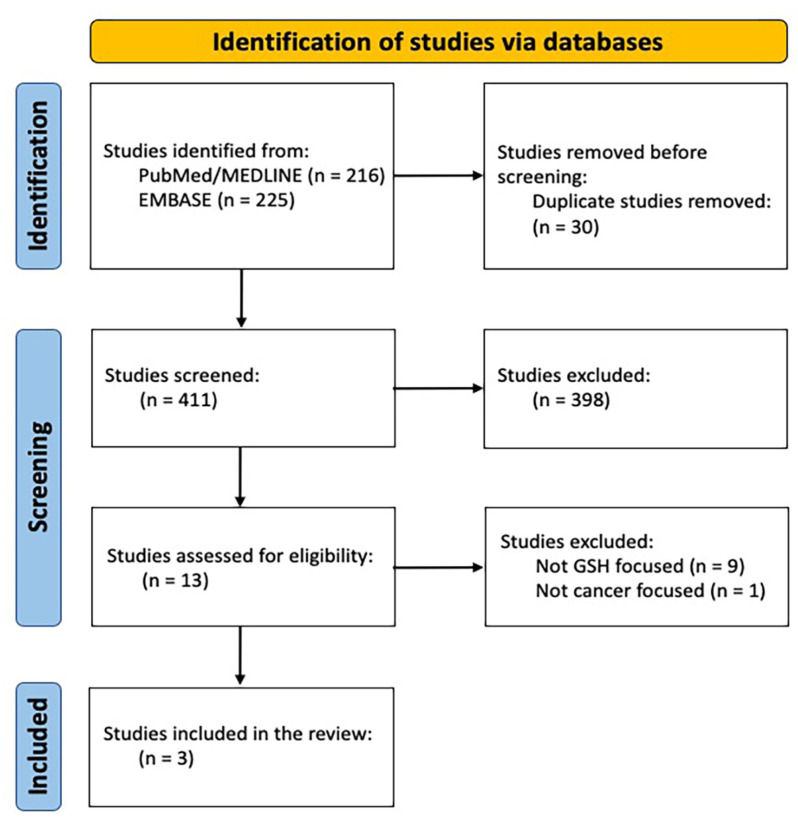
PRISMA diagram [3].

In order to evaluate the role of GSH and its antioxidant effects in patients affected by cancer, we performed a thorough search on the Medline and EMBASE databases, focusing on clinical and/or preclinical studies. Full texts of included papers written entirely in English with available abstracts and at least one of the following characteristics were considered: clinical and/or preclinical studies on the role of GSH in cancer patients, with particular regard to antioxidants’ effects, diet, toxicities, and pharmacological processes, including pharmacokinetic aspects and risk of interactions. Boolean operators AND/OR were used to combine search terms. The following search strings were used in PubMed: ((((“Glutathione”[Title/Abstract]) OR (“GSH”[Title/Abstract])) AND ((((“Cancer”[Title/Abstract]) OR (“Carcinoma”[Title/Abstract])) OR (“Tumor”[Title/Abstract])) OR (“Tumour”[Title/Abstract]))) AND (((“Diet”[Title/Abstract]) OR (“Dietary”[Title/Abstract])) OR (“Nutrition”[Title/Abstract]))) AND ((“Toxicity”[Title/Abstract]) OR (“Toxic”[Title/Abstract])). The databases were last accessed on 28 February 2023. HRM, BAF, LM and MB searched articles published in English by February 2023, chosen according to inclusion and exclusion criteria. We created a Preferred Reporting Items for Systematic Reviews and Meta-analyses (PRISMA) flow diagram [3] on 20 March 2023, to summarize the process followed in the systematic review. Figure 1 shows the results of the review based on the PRISMA guidelines [3]. 

An overview of the metabolism, dietary implementation, and role of GSH in both healthy and cancerous cells, as well as its possible effects on the toxicities and the efficacy of anticancer treatments was also displayed to completely understand the role of GSH in cancer treatment. 

## 3. A Quick Overview of GSH Metabolic Processes

Under conditions of cell injury, an increased production of ROS is observed, despite their normal presence in healthy cells [4,5]. In fact, a physiological ROS level intervenes in essential signaling pathways and it is vital for cell survival. On the contrary, excessive ROS levels cause cell injury by damaging proteins, lipids, and nucleic acids, leading to different conditions, such as neurological diseases, hypertension, diabetes, atherosclerosis, heart failure, chronic kidney disease, and cancerogenesis [2]. As a consequence, adaptive responses, such as the synthesis of GSH, are necessary to re-establish the redox homeostasis. However, GSH is also involved in the catabolism of toxins, xenobiotics, and drugs, in the regulation of immunity [6], in the synthesis of leukotrienes and prostaglandins [7], in the regulation of the metabolism of many vitamins [8], in glutamate neurotransmitter metabolism [9] and neural regulation [10], and in embryonic development [11].

**Figure 2:**
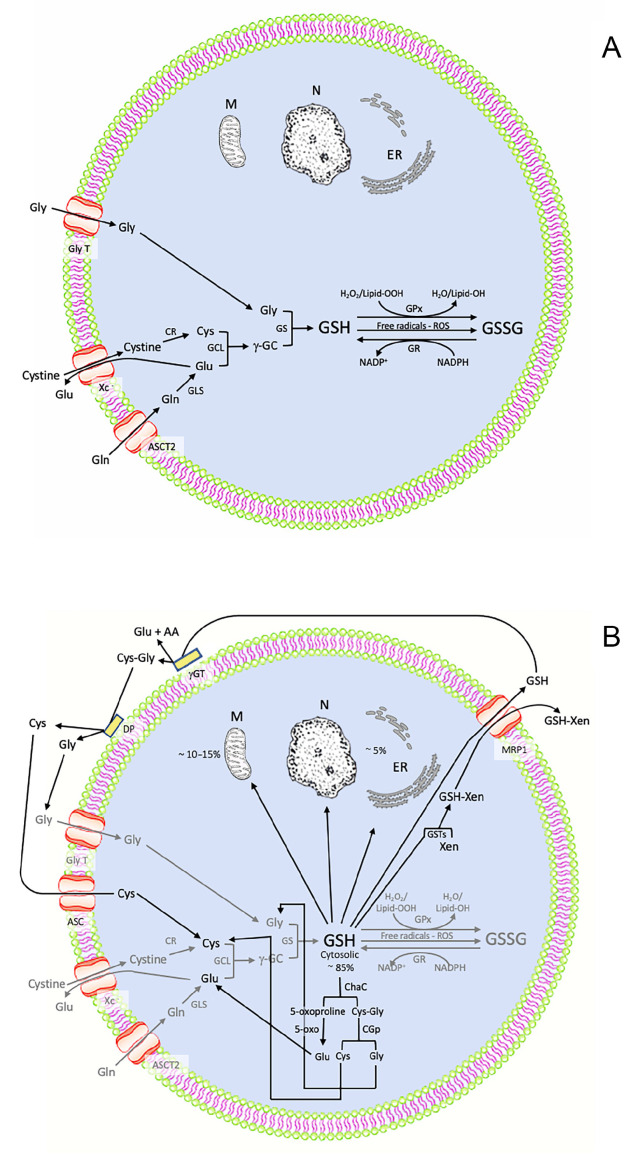
(**A**) Biosynthetic pathway and antioxidant role of GSH and its interconversion into the oxidized form GSSG. (**B**) Intracellular distribution, degradation pathways, and recycling of GSH. GSH = glutathione; N = nucleus; M = mitochondrion; ER = endoplasmic reticulum; Gln = glutamine; Glu = glutamic acid; Cys = cysteine; Gly = glycine; Gly T = glycine transporter; Xc- = cystine/glutamate antiporter; ASCT2 = Alanine/serine/cysteine transporter 2; GLS = glutaminase; γ-GC = γ -glutamyl-cysteine; GCL = glutamate-cysteine ligase; GS = glutathione-synthetase; GPx = glutathione peroxidase; GSSG = glutathione disulfide; GR = glutathione reductase; Cys-Gly = cysteine–glycine; ASC = Alanine/serine/cysteine transporter 1; MRP1 = multidrug-resistant protein 1; GSTs = glutathione S-transferases; Xen = xenobiotics; GSH-Xen = glutathione plus xenobiotics; γ-GT = γ-glutamyl transferase; DP = Cysteinyl glycine dipeptidase; ChaC = glutathione-specific gamma-glutamylcyclotransferase; CGp = Cys-Gly peptidase; 5 oxo = 5-oxoprolinase.

GSH is synthesized in the cellular cytosol from its precursor amino acids Glu, Cys, and Gly, which are brought into the cell by specific transporters (Figure 2A) [12,13,14]. Once in the cytosol, Glu and Cys are combined into the dipeptide γ-glutamylcysteine (γ-GC) by the enzymatic activity of glutamate–cysteine ligase (GCL), which requires ATP and Mg^2+^ as co-substrates (Figure 2A) [15,16,17]. This step is particularly important as Glu and Cys are linked by a stable γ-peptide bond instead of the typical α-peptide linkage, thus providing a stronger resistance to the action of most intracellular enzymes [18]. Furthermore, Cys with its redox active thiol (SH) ensures the reducing characteristics of GSH [18]. Then, γ-GC is combined with Gly through a typical α-linkage by the enzyme GSH synthetase (GS), which also requires ATP and Mg^2+^ as co-substrates, ultimately resulting in the formation of GSH (Figure 2A) [11]. It is therefore possible to state that GSH synthesis is based on the availability of basic resources (amino acids), energy (ATP), and enzymes (GCL and GS) [19]. 

GSH is present mainly (~85%) in the cytoplasm at a millimolar concentration and then distributed into mitochondria (~10–15%) and endoplasmic reticulum and nucleus (~5%) (Figure 2B) [17]. Under oxidative stress, two molecules of GSH react, forming the oxidized GSH disulphide (GSSG) (Figure 2A) [19], which can be reconverted to its reduced form (GSH) by glutathione reductase (GR). Cytosolic GSH can be enzymatically degraded into the cell [20,21], so that Glu, Cys, and Gly can be reused for the synthesis of new GSH (Figure 2B) [21].

Some cytosolic GSH is transported, thanks to a specific transporter, the multidrug resistance protein 1 (MRP1), out of the cell to maintain a balance in the redox level [22], or can be conjugated by GSH transferases (GST) with endogenous metabolites, such as cysteinyl leukotriene or xenobiotics (therapeutics, anticancer drugs, carcinogens, pesticides, herbicides), forming a complex GSH-Xen, which is transported outside the cell by MRP1 [19].

Extracellular GSH is degraded by the enzymes γ-glutamyl transpeptidase (γ-GT) and dipeptidases (DP) into its structural amino acids (Figure 2B), which, once into the cell, are reused for GSH synthesis [17]. In this manner, the “GSH cycle” takes place (Figure 2B), which includes the synthesis, cellular distribution, and metabolism of the molecule [21]. 

### 3.1. Genetic Findings of GSH

GS deficiency is a rare disorder of glutathione (GSH) metabolism manifested in children by severe metabolic acidosis with high urinary level of 5-oxoproline (pyroglutamic acid), hemolytic anemia, and neurological events. The disorder has severe to moderate clinical variants [23]. 

Furthermore, no well-defined genetic variants were found in GSH, except for single nucleotide polymorphisms (SNPs) in GS, namely rs7265992, rs6060124, rs7260770, rs4911455, rs6088662, and in GPX5, namely rs377514. These SNPs have been linked to bladder cancer [24].

On the contrary, parental GST genes, namely GSTM1, GSTT1, and GSTP1, have been extensively studied with respect to polymorphisms. The main allelic variants of the GST gene that lead to significant changes in protein expression are GSTP1 C341T→A114V, GSTT1 null/null genotypes, and GSTM1 and GSTM3 (rs11101992 CC), (rs1571858 GG + GA), and GSTM4 (rs560018 GG). Specifically, GSTP1 results in reduced catalytic activity, while GSTT1 and GSTM1 null/null genotypes exhibit a complete absence of catalytic activity.

In addition, extensive study of neoplastic tissues by 5′UTR CG-methylated islands analysis show the inactivation of the GSTP1, due to the hypermethylation. This feature could be a potential biomarker for several types of cancer. This specific and sensitive detection method for assessing the methylation status of the GSTP1 gene shows great promise as a rapid screening test to determine risk factors through a simple blood test [25].

### 3.2. GSH Role in Cancer Cells

It was demonstrated that in cancer, GSH may play a double role according to the different stages (initiation and progression) [19]. In fact, during initiation, GSH counteracts ROS-induced DNA oxidation and DNA damage through a mechanism of elimination of carcinogens by the GSTs [26]. During the progressive stage of cancer, intracellular GSH rates are upregulated to oppose the considerable ROS, thus protecting cancer cells from apoptosis induced by oxidative stress [27]. 

In cancer cells, the oxidative stress is particularly elevated, so that GSH depletion could make them more susceptible to therapy [28]. In fact, when ROS levels are strategically increased, cancer therapy has shown better results, even if the presence of biological barriers could limit their efficacy. Therefore, one of the therapeutic strategies in cancer should be considered the depletion of the cellular antioxidative systems, among which GSH is one of the main actors [19]. However, even if the requests in clinical treatments of cancer are continuously in progress, new studies are necessary to better explain the relationship between GSH and cancer.

Recent studies have demonstrated that the depletion of GSH can be obtained acting on the different steps of the “GSH cycle”, triggering mechanisms able to stimulate, as a final result, cancer cell death. 

This can be obtained through a non-apoptotic process of regulated cell death termed ferroptosis [29]. It is largely initiated by extra-mitochondrial lipid peroxidation occurring from an iron-dependent ROS accumulation, caused by abnormal iron metabolism or disturbances in the main redox systems, lipid peroxidation, and thiols, in particular GSH [30]. As a result, an accumulation of Lipid-OOH occurs: since GSH is the reducing co-substrate of GPX4, able to regulate the redox state of the cell-forming Lipid-OH (Figure 2A), GSH depletion can negatively regulate GPX4, triggering ferroptosis [19]. This process can be also elicited modulating cellular sensitivity through the cystine/glutamate antiporter (system Xc-) suppression, acting on their intracellular concentrations, or reducing the activity of GLS2, the enzyme catalyzing the conversion of glutamine into glutamate [31].

When the effects of antineoplastic drugs are considered, it was demonstrated that the treatment with doxorubicin (DOX), an anticancer drug belonging to the anthracycline class, lowered GSH levels in cancer cells, and that exogenous GSH could increase cytosolic GSH levels, thus highly reducing collateral effects of DOX, such as cardiotoxicity and hepatotoxicity; in fact, in experimental models, GSH avoided the inotropic effects and lowered transaminases in DOX treatment. However, it was also suggested that the association DOX–GSH significantly decreased the antineoplastic effectiveness of DOX in a dose-dependent manner, thus proving a disadvantageous effect of GSH use [32].

Another effect of GSH depletion could be related to the action of GSTs on antineoplastic drugs [33]. In fact, an increase in the expression of GSTs is involved in drug resistance. It enhances the conjugation of GSH with different substances, including some chemotherapeutic drugs, such as chlorambucil (CBL) or cisplatin. CBL, a nitrogen mustard alkylating drug, shows a different behavior in the presence or absence of GSH. When GSH is present, CBL is conjugated with GSH by the induction on the specific transferase hGSTA1-1 [34], thus inducing an efflux from the cells and reducing CBL activity. On the contrary, GSH absence inhibits hGSTA1-1 activity, thus increasing CBL efficacy. Moreover, the resistance to CBL is not solely attributed to the involvement of GSTA1-1, but rather requires the combined expression of MRP1 or MRP2 as well [34].

Regarding cisplatin, which belongs to the class of alkylating agents, it has the ability to bind with intracellular GSH through the enzymatic action of GST. Subsequently, cancer cells can eliminate it [35]. In particular, the chemotherapeutic response and the vulnerability to cisplatin depends on GSTP1, able to induce the conjugation of GSH to electrophilic molecules. Similar mechanisms were described also for oxaliplatinum. In fact, the presence of a well-known genetic polymorphism of GSTP1 (A313G→I105V) was associated with the occurrence of side effects, such as peripheral neuropathy observed in patients with colon cancer. In this way, GSTP1 genotype could be considered a prognostic marker, able to reduce or avoid the peripheral neuropathy [36]. On the contrary, some reports demonstrated that high doses of exogenous GSH-reduced cisplatin induce neurotoxicity [37]. Several studies report that an important role in taxanes’ toxicity is played by ROS both in vivo and in vitro [38]. These molecules can damage almost all the structures inside cells and, since their high harmful potential, different protective mechanisms exist. Among these, the GSTs system is widely used by cells to detoxify ROS and several other toxic molecules. Regarding taxanes pharmacogenomics, it is important to report that polymorphisms of this gene are strongly related to increased toxicity. All these variants are associated not only with an increased risk of developing certain types of cancers (i.e., colorectal), but also with a greater susceptibility in developing severe toxicities (≥grade 3) during different chemotherapy regimens, including those containing taxane.

Among the mechanisms of GSH conjugation with anticancer drugs, GSH S-conjugates are formed through the mechanism called Michael addition or conjugate addition [39]. This occurs when α,β-unsaturated compounds with double or triple carbon–carbon bonds are added to a soft nucleophile, such as the thiol group of cysteine of GSH [16]. The resulting compounds are particularly reactive and are able to bond with biological macromolecules. Examples of compounds that undergo GSH conjugation by Michael addition are mitoxantrone, a synthetic anthraquinone antineoplastic agent; mitomycin C, a natural cytostatic antibiotic; and infigratinib, afatinib, ibrutinib, and neratinib, inhibitors of various tyrosine kinases [16].

## 4. Glutathione, Nutrition and Nutraceuticals

GSH is a biological tool related to risk of a disease and well-being status; therefore, the optimization of GSH levels has been proposed as a preventive and therapeutic strategy against diseases including cancer.

**Figure 3:**
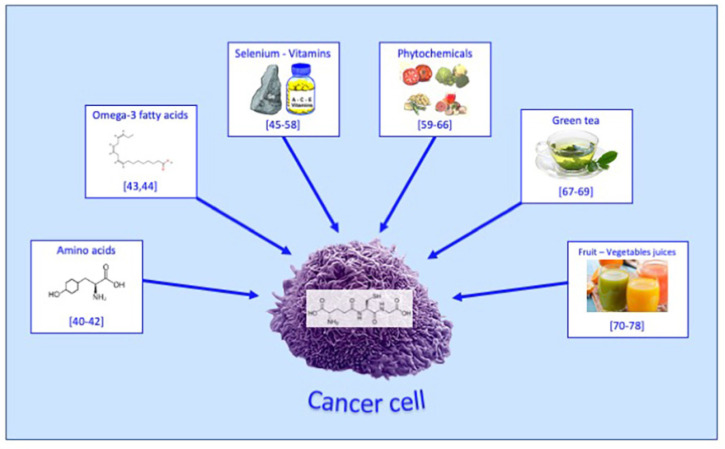
Bioactive compounds from diet able to interfere on cancer cells by modulating circulating GSH levels. Amino acids: see references [40,41,42]; omega-3 fatty acids: see references [43,44]; selenium-vitamins: see references [45,46,47,48,49,50,51,52,53,54,55,56,57,58]; phytochemicals: see references [59,60,61,62,63,64,65,66]; green tea: see references [67,68,69]; fruit-vegetables juices: see references [70,71,72,73,74,75,76,77,78].

Indeed, in recent years, human clinical research has indicated that nutritional interventions, such as (a) amino acids, (b) omega-3 fatty acids, (c) minerals (i.e., selenium) and vitamins, (d) phytonutrients, (e) green tea, (f) fruit and/or vegetables (i.e., juices), can significantly influence circulating GSH levels. These changes in GSH levels have the potential to yield clinical benefits, as illustrated in Figure 3.

In this context, it is crucial to underline the difficulty in clearly identifying how to monitor GSH status, as well as it not being easy to establish the influence of genetic variations and responses to nutritional factors able to influence GSH status. Another critical issue is related to the identification of optimal doses, delivery forms, and bioavailability of these bioactive compounds in cancer patients. 

### 4.1. Amino Acids

An adequate intake of dietary proteins may affect the amino acid pool from which GSH is synthesized. Consequently, changes in protein consumption [40], as well as an impaired protein digestion, may decrease plasma GSH levels, potentially contributing to a reduction in total antioxidant capacity of human body.

To support this hypothesis, a randomized double-blind controlled trial conducted in 2018 suggested that in cancer patients, consuming 40 g of whey protein isolates, in addition to zinc and selenium (Se), significantly increased GSH levels, as well as functional immune markers [40].

Interestingly, serine may also positively support the GSH production in experimental animal studies, probably increasing cysteine availability and reducing hypermethylation [41,42].

### 4.2. Omega-3 Fatty Acids

Gago-Dominguez and co-workers [43] investigated the effect of GST polymorphisms on the relationship between omega-3 fatty acids and breast cancer risk in post-menopausal women. Specifically, they showed a significant increased protective effect of a dietary intake of marine-based omega-3 fatty acids in Chinese women with genetic polymorphisms. 

Recently, however, Sepidarkish and colleagues showed that the co-supplementation of omega-3 fatty acids with vitamin E was not able to induce any significant changes in GSH concentrations nor in superoxide dismutase and catalase levels [44]. Overall, these data suggest that further investigation is still required to assess the benefits of omega-3 fatty acids’ supplementation in cancer-associated illnesses. Nevertheless, since omega-3 fatty acids and their metabolites might modulate pivotal molecular pathways including metabolic routes for the three amino acids (cysteine, glycine, and glutamate) needed for GSH biosynthesis, this feature could represent a promising field of knowledge to be explored.

### 4.3. Selenium and Vitamins 

Selenium (Se) is an essential bio element, necessary for the correct functioning of all organisms; it is also available in multivitamin/multimineral supplements and as a standalone supplement [45,46]. 

Se, through GSH reductase and other selenoproteins, regulates the antioxidant activity in cells, thus protecting the human body against harmful effect of free radicals and, consequently, reducing the risk of cancer [47,48]. Individuals with low serum Se level together with vitamin E deficiencies, are at an increased risk of developing cancer [49]. Moreover, in healthy subjects, the blood level of Se is significantly higher in comparison with cancer patients [50]. Both organic and inorganic Se compounds (including selenoglutathione), ingested with the common diet, significantly reduced chemically induced cancers [51]. 

The amount of this trace element can be different; indeed, plant foods are the major dietary sources of Se and its amount in diet depends by several factors, such as soil Se concentrations, soil pH, geographic location, types and amounts of food consumed, etc. [52,53]. Moreover, protein-rich foods were found to contain higher levels of Se, whereas low levels were found in plants with low protein content. 

So far, cereals, meat, dairy products, fishes, seafood, milk, and nuts are particularly rich in Se [54]; similarly, Se is present in sea salt, bread, mushrooms, garlic, and asparagus [55,56]. Its bioavailability is reduced by potentially toxic elements (PTEs) and sulfur, but it increases in the presence of vitamins A, C, E, and proteins containing methionine [55,57]. Interestingly, a protective role of Se occurs in organs that are the typical body sites of human cancers (i.e., stomach, intestine, mammary glands, and liver) [47]. Thus, current research is focused on obtaining plants enriched in Se and, consequently, on the possibility of obtaining nutraceuticals/functional foods from them [58], particularly acting on the metabolic GSH-related pathways in human body. 

### 4.4. Phytochemicals

Conflicting results from supplementation with different doses of antioxidant vitamins and minerals in isolation and or separate from their phytonutrient complements are shown in clinical studies. One reason to explain these discrepancies may derive from the fluctuations in the redox state of a cell [59]. Therefore, a rational dietary approach to positively regulate the innate defense system against oxidative stress also improving the GSH status in cells may be obtained by choosing diversity from a healthy nutritional point of view, then strategically promoting consumption of multiple phytonutrients. In this regard, the effect of fruit and vegetable intake on GSH and GSH-related enzyme levels has been studied, indicating that GST activity is related with cancer prevention [60,61]. 

Indeed, several studies by different research groups suggest the detoxification and cancer-preventive qualities of cruciferous vegetable intake [62,63] especially for cancers of the gastrointestinal tract [64]. Other experimental studies in vivo [65,66] have shown that GSH molecular pathways are increased, although not always significantly, after sulforaphane, a cruciferous-derived compound, administration [65]. Overall, cruciferous and cruciferous-derived compounds may play a crucial role in individuals with GST polymorphisms; however, their contribution to cancer management remains to be evaluated.

### 4.5. Green Tea 

Green tea consumption is associated with reduced rates of certain cancers such as leukemia [67].

In fact, Liu et al. demonstrated an inverse association between drinking green tea and adult leukemia risk; moreover, cancer risk reduction was more elevated in patients with the glutathione S-transferase theta 1 (GSTT1)-null genotype than the GSTT1-present carriers [67]. However, there is no clear evidence that green tea can have a positive role in cancer therapy. Additionally, green tea extracts were demonstrated to be potent inhibitors of CYP 450 [68] and this feature indicates extreme caution in cancer patients receiving chemotherapy.

Overall, the results are inconclusive due to several factors. Firstly, clinical studies often lack an adequate number of subjects to draw definitive conclusions. Additionally, variations in the dosage of tea administered further contribute to the uncertainty. Furthermore, important factors such as genotype and lifestyle have not been taken into consideration, which may influence the outcomes. Consequently, further large-scale clinical trials are required to ascertain the potential of green tea in the prevention or treatment of cancer [69].

### 4.6. Fruit and/or Vegetable Juices

Drinking juices derived from fruit and/or vegetables may provide another healthy option, probably related to their simple sugar amount. According to currently available clinical studies, drinking fruit and/or vegetable juices improves antioxidant status. A reduction in oxidative stress can be obtained by the assumption of polyphenol-rich juices such as pomegranate and grape juice [70,71,72]. Intriguingly, the intake of purple grape juice for 8 weeks positively affected diastolic blood pressures, DNA damage and antioxidant status in smokers, mainly greater in Glutathione S-Transferase Mu 1 (GSTM1)-null and GSTT1-present types. One of these studies further confirmed the crucial role of genotype in the regulation of GSH metabolism in humans [71].

Finally, several animal studies indicated that some herbs and roots, such as rosemary [73,74,75], turmeric/curcumin [76], milk thistle [77], and gingko biloba [78] may significantly increase GSH levels.

### 4.7. Mediterranean Diet, Nutraceuticals and GSH: Perspectives and Challenges

Several foods contain thiol-rich compounds, including GSH. Consequently, eating a GSH-supported diet could include these foods daily, especially green foods, asparagus, avocado, cucumber, green beans, and spinach. Intriguingly, feeding mice with a Western-style diet both reduced GSH synthesis in liver and its levels in plasma [79]; moreover, in humans a higher compliance to a Mediterranean-style diet is related to higher plasma GSH [80]. Then, exploring food-based nutritional interventions specifically tailored to optimize GSH levels could be a potential therapeutic strategy [81]; the plant-based diets, such as the Mediterranean diet, have demonstrated possible protective effects against non-communicable diseases, including cancers [82,83]. The Mediterranean diet is currently recognized as an effective strategy in combating cancer due to its ability to provide protection against oxidative and inflammatory processes in cells. As a matter of fact, this protective effect is achieved through the regulation of GSH metabolic pathways, prevention of DNA damage, control of cell proliferation and survival, as well as inhibition of angiogenesis and metastasis [84,85]. Some multiple molecular mechanisms of biological components obtained from plants are directed towards hallmarks of cancer. The Mediterranean diet is particularly rich in nutraceuticals which can be used in different fields, such as food, cosmetics, and drugs [86]. In this context, improving dietary intake of GSH could represent a relatively attractive, simple, low cost, and safe [81] strategy against cancer.

On the basis of this background, the introduction into the diet of foods enhancing GSH status, such as lean protein sources, brassica vegetables, fruits and vegetables rich in polyphenols, herbs and spices, green tea, and fish, is highly recommended. 

## 5. Glutathione and Cancer

Glutathione and its biologically active reduced form (GSH), as stated above, is a molecule that can be found inside the cells of many different organisms, including animals, fungi and plants [87,88]. In human beings, this substance is widely distributed in the various organs and tissues, with the maximum concentration in the liver, followed by the spleen and kidneys. It plays a key antioxidant role in human cells, thus its homeostasis appears fundamental to the wellbeing of every tissue [89]. Given the well-known protective effect of GSH against potentially harmful substances, this molecule has for a long time been widely used, after medical prescription or as self-medication, to minimize toxicities induced by several anti-cancer treatments, especially chemotherapy. However, patients have limited knowledge regarding the potential detrimental effects that may arise from the implementation of this treatment during their oncologic treatment.

The conjugation of GSH with xenobiotics including anticancer drugs can, indeed, lead to a double effect: on the one hand, xenobiotics might lose their damaging effect [90]; on the other hand, GSH conjugation might enhance their toxicity inducing their bioactivation [91]. 

Although non-enzymatic GSH conjugation reactions are possible, the majority of these reactions are catalyzed by GSH-dependent enzymes, particularly GSH S-transferase [92]. Through these reactions, GSH can combine with anticancer drugs or their metabolites to generate conjugates that are less toxic or more easily excreted [90]. On one hand, this role reduces the toxic effects that anticancer drugs may have on healthy non-cancerous cells, but on the other hand, it may also diminish their effectiveness on cancerous cells, thereby promoting the early onset of anti-cancer drug resistance [1,17,93,94,95].

As stated above, GSH conjugation can also lead to the generation of more active compounds that may be even more pharmacologically active and toxic than the originator molecule [91]. Busulfan toxicity, for instance, has been related to its GSH-conjugate metabolites. Its well-known adverse events, e.g., interstitial pulmonary fibrosis, seizures, liver veno-occlusive disease, or sinusoidal obstruction syndrome [96] seem to be caused by glutathionyl-tetrahydrothiophene, a metabolite that derives from the irreversible GSH conjugation [97,98].

Bioactivation reactions mediated by GSH, besides enhancing some drugs’ toxicities, might also represent the fundamental step in generating some pro-drugs active metabolites. Azathioprine, for example, necessitates GSH conjugation to be converted in its active metabolite 6- mercaptopurine, the actual effector of its anti-cancer purpose [99,100]. The cisplatin bioactivating effect will be discussed further on in this paper.

### 5.1. GSH and Platin-Compounds Related Toxicities

Cis-diaminodichloroplatinum, also known as cisplatin or Cis-DDP, was one of the first metals to ever be used in oncological clinical practice and still represents one of the cornerstone drugs in the treatment of several cancers, including lung, testis, head and neck and gynecological cancers. 

It carries out its cytotoxic action by interacting with DNA and inducing direct damage that leads the cell to apoptosis [101].

As with the majority of anticancer drugs, its cytotoxic action comes along with possible damage for different non-neoplastic tissues, inducing toxicities that may affect patients’ quality of life. Damage to peripheral sensitive nerves occurs through a mechanism that is still not fully understood. For example, it is the underlying cause of the well-known neurotoxicity induced by cisplatin (Cis-DDP), which often leads to the discontinuation of chemotherapy based on Cis-DDP. The clinical presentation of this condition is diverse, and the intensity of symptoms typically corresponds to the severity of nerve damage. Mild forms may manifest as distal paresthesia, while severe neuropathies can result in sensory ataxia. Neurotoxicity is apparently dose-dependent (frequent for 250–350 mg/m^2^, almost certain if dosage exceeds 500–600 mg/m^2^) [102], although cases of early onset have been described, particularly in patients with neuropathy risk factors [103]. 

To reduce Cis-DDP neurotoxicity incidence and severity, the coadministration of several neuroprotective agents has been studied, including GSH [104]. GSH, in particular, would act as a neuroprotective agent binding Cis-DDP and other platinum compounds such as oxaliplatin, preventing the first step of neuronal damage that is platinum accumulation in peripheral nerves [105,106].

A recent meta-analysis by Alberts et al. [104] evaluated GSH’s effect on neurotoxicity during oxaliplatin- and cisplatin-based chemotherapy regimens [105,106,107,108,109,110,111]. A reduction in peripheral neuropathy in the GSH-implementation arms was demonstrated in different neoplasms (head and neck cancer, non-small cell lung cancer, ovarian cancer, gastric cancer, colorectal cancer), suggesting a possible neuroprotective effect of this substance. Larger trials conducted on a broader population would be valuable in confirming these findings and establishing GSH as a neuroprotective agent in clinical practice.

Although several pieces of data suggest a possible therapeutic effect of GSH, administered directly or through its precursor N-Acetil Cystein (NAC), in drug-induced liver disease (DILI) [112,113], no randomized-controlled clinical trials are available to support this evidence. For this reason, GSH implementation should not be used in drug-induced toxicities different than platinum-related neurotoxicity, that at this moment represents its only clinical indication.

Besides its possible neurotoxicity-reducing action, GSH seems also involved on the other hand in the worsening of other Cis-DDP-related toxicities. Cis-DDP’s well-known nephrotoxicity, indeed, has been proved to be related to his GSH-conjugated compounds that, being metabolized to cysteine S-conjugate by cysteine S-conjugate β-lyase activity (highly expressed in renal cells), is mainly responsible for proximal tubule cell damage [114]. 

### 5.2. GSH Favourable Effects on Cancer Cells

While being an interesting weapon against chemotherapy-induced toxicities, it is mandatory to assess that GSH, as an anti-oxidative agent, might have a potential protective role on cancer cells, reducing anticancer treatment efficacy. This seems mediated not only by the reduction in ROS accumulation and the subsequent DNA damage, but also by the direct bond between GSH and drugs that may favor their excretion or may lead to non-effective compounds [115]. Tumor cells, indeed, have shown higher levels of GSH than normal cells and often are characterized by an overexpression of GSH-related enzymes and proteins that lead to a better resistance to antineoplastic treatments and, possibly, could favor chemoresistance [116,117,118]. For instance, an inverse relationship was described between the chlorambucil effect and GSH levels and activity [119,120] or between GSH levels and the concentration of cyclophosphamide active metabolites, suggesting a possible decrease in their efficacy during anticancer treatments [121]. 

Therefore, GSH depletion has always caught researchers’ attention but has not led to cutting-edge results [122,123]. We have already discussed the fundamental role of GSH in avoiding ROS accumulation leading to cancerous transformation, as well as its crucial role in tumor cells’ survival. Besides these effects, GSH’s increase has demonstrated the induction of proliferation both in normal and malignant cells through a still undefined mechanism [124,125]. This activity was also confirmed in vivo in mice inoculated with B-16 melanoma. When the inoculation took place in the presence of high levels of GSH, both the number and size of metastases appeared to be higher [126,127].

## 6. Discussion

Of the 441 articles that resulted from our research, 30 were excluded by automatic tools as duplicates. Of the 411 resulting articles screened by full text and abstract, 398 were excluded; 13 articles were assessed for eligibility and 3 were eventually included in the analysis. The three selected articles [122,127,128] are analyzed hereafter. 

In the first article by Wolff C.M. et al., “Synergistic In Vitro Anticancer Toxicity of Pulsed Electric Fields and Glutathione” [128], the authors display their analysis of the potential synergistic in vitro activity between pulsed electric field (PEF) and glutathione against skin cancer cells. Although being a preclinical study that might not imply a strict correspondence with in vivo results, their strategy of comparing different treatments (e.g., GSH alone vs. PEF alone vs. GSH combined with PEF) and of different types of skin cancer cells (A375 and MNT-1 for malignant melanoma cells and A431 and SSC-25 for squamous skin cell carcinoma) increases their work reliability. The decrease in viability of all the skin cancer cell lines except for SCC-25 cells implies a possible role of this combination treatment in this type of cancers, although the SCC-25 cells’ intrinsic resistance should be deepened. Clinical studies are necessary to confirm the preclinical data. 

In the original research published in 2007 by Mena S. et al., “Bcl-2 and Glutathione Depletion Sensitizes B16 Melanoma to Combination Therapy and Eliminates Metastatic Disease” [122], the in vivo effects of the combination of GSH and Bcl-2 depletion with various anticancer treatments (such as paclitaxel, X-rays, tumor necrosis factor-α, and IFN-γ) were examined. Murine models were inoculated with murine B16 melanoma (B16M) to obtain in vivo metastatic melanoma that was subsequently tested with the above-stated combination treatments. Despite the promising results of the experiment, which demonstrated long-term survival (>120 days) without recurrence in 90% of mice that received the complete combination treatment, this therapeutic approach may not be suitable in the era of immune checkpoint inhibitors, which began in 2010. Another key point to assess is that murine models treated in this study were affected by liver-only metastases, with the liver being a particularly GSH-rich organ, that may represent a bias for evaluating this combination as an effective response in clinical practice, considering that melanoma metastases may occur in every other organ. 

The paper “Antioxidants Can Increase Melanoma Metastasis in Mice” [127] published by Kristell Le Gal et al. in 2015 exposes a possible cancer-stimulating effect of antioxidants, particularly NAC, that, as stated above, represents a GSH precursor, on in vivo and in vitro melanoma models. They did not observe an increased proliferation of melanoma cells upon NAC implementation. However, there was an increased tendency for migration and invasive behavior. In light of the contradictory findings presented by the authors in comparison to previous research, it is evident that further studies are warranted.

**Figure 4:**
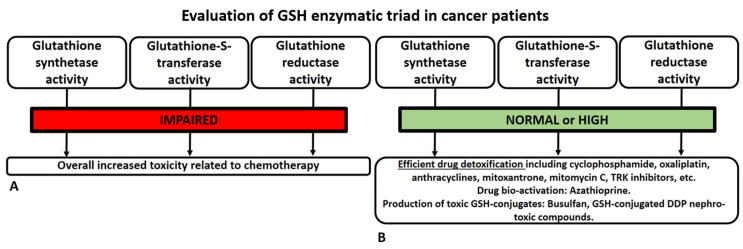
A rational use of GSH in cancer patients should not overlook an ideal and minimal evaluation of the enzymatic triad involved in GSH synthesis and metabolism (see polymorphisms, expression, and epigenetic control discussed in the manuscript). Indeed, there may be different activity levels of the enzymatic triad in the patient and the tumor, with indirectly assessable effects. When one of the enzymes has reduced activity, it affects the overall GSH production balance, leading to a decrease in its concentration (and corresponding antioxidant power). The scenario where there is reduced activity in cancer patients, in general, may be associated with increased chemotherapy efficacy, albeit at the cost of greater toxicity for the patient (**A**). In cases where the enzymatic triad functions well in healthy cells but is impaired in the tumor cells, it represents a positive scenario where chemotherapy can have a greater damaging effect on the neoplasm. However, as shown in the last panel (**B**), this is not true for all drugs. It would be desirable to further investigate the biology of cancer regarding these aspects, especially when the enzymatic triad is “overactive” only in the tumor. Such an approach could lead to the pharmacological design of specific inhibitors targeting the tumor enzymes involved in GSH metabolism.

Although many publications dating back to the beginning of this century about antioxidants in general (and GSH in particular) only tend to underline the beneficial effects that these substances have in cancer patients, possibly preventing cancer onset and curing anticancer treatment adverse events, much more has been discovered in recent years, as widely displayed in this review. For this reason, GSH administration should be limited to its approved indications (e.g., platin-induced neurotoxicity) or to clinical/preclinical studies where applicable. The risk of self-prescription by cancer patients is high, especially if the GSH is used as alternative treatment [129,130,131,132]. An ideal and future approach to the use of GSH in cancer patients is depicted in Figure 4. 

## 7. Conclusions and Perspectives

GSH certainly represents a powerful antioxidant agent, fundamental for normal cells’ homeostasis and survival. Its adequate levels seem to play a key role in the prevention of different types of cancer. This common knowledge has increasingly led to the diffusion of self-prescribed GSH implementation by cancer patients, aiming to reduce anticancer treatments’ toxicities and possibly prevent damage to normal tissues. This belief is true if cisplatin-related neurotoxicity is considered, while it lacks solid background evidence for every other toxicity. For this reason, GSH should not be used by patients as self-medication and should only be assumed on medical prescription for the appropriate time and setting. The GSH protective effect on cancer cells might indeed contrast anticancer drugs’ efficacy while not offering a solution to patients’ toxicities. 

When searching for the keyword “Glutathione” on Clinicaltrial.gov, a total of 106 currently recruiting clinical trials are found. These trials primarily investigate the antioxidant effect of glutathione in healthy individuals or various diseases. However, when narrowing down the search to include “cancer,” the results decrease to 12 trials. None of these trials specifically analyze the implementation of GSH alone or in combination with other drugs. Considering the multiple effects of this powerful antioxidant agent and its interactions with cancer cells and anticancer treatments, further studies are certainly needed to define once and for all its role for cancer patients. 

## Data Availability

No new data were created or analyzed in this study. Data sharing is not applicable to this article.
